# HMGB1 Promotes Systemic Lupus Erythematosus by Enhancing Macrophage Inflammatory Response

**DOI:** 10.1155/2015/946748

**Published:** 2015-05-19

**Authors:** Mudan Lu, Shanshan Yu, Wei Xu, Bo Gao, Sidong Xiong

**Affiliations:** ^1^Institute for Immunobiology and Department of Immunology, Shanghai Medical College, Fudan University, Shanghai 200032, China; ^2^Jiangsu Key Laboratory of Infection and Immunity, Institutes of Biology and Medical Sciences, Soochow University, Suzhou 215123, China

## Abstract

*Background/Purpose*. HMGB1, which may act as a proinflammatory mediator, has been proposed to contribute to the pathogenesis of multiple chronic inflammatory and autoimmune diseases including systemic lupus erythematosus (SLE); however, the precise mechanism of HMGB1 in the pathogenic process of SLE remains obscure. *Method*. The expression of HMGB1 was measured by ELISA and western blot. The ELISA was also applied to detect proinflammatory cytokines levels. Furthermore, nephritic pathology was evaluated by H&E staining of renal tissues. *Results*. In this study, we found that HMGB1 levels were significantly increased and correlated with SLE disease activity in both clinical patients and murine model. Furthermore, gain- and loss-of-function analysis showed that HMGB1 exacerbated the severity of SLE. Of note, the HMGB1 levels were found to be associated with the levels of proinflammatory cytokines such as TNF-*α* and IL-6 in SLE patients. Further study demonstrated that increased HMGB1 expression deteriorated the severity of SLE via enhancing macrophage inflammatory response. Moreover, we found that receptor of advanced glycation end products played a critical role in HMGB1-mediated macrophage inflammatory response. *Conclusion*. These findings suggested that HMGB1 might be a risk factor for SLE, and manipulation of HMGB1 signaling might provide a therapeutic strategy for SLE.

## 1. Introduction

Systemic lupus erythematosus (SLE) is an autoimmune disease characterized by chronic inflammation in multiple organs such as kidney, lung, heart, joint, and so forth [1–5]. One of the most severe manifestations of SLE is lupus nephritis, which is a potentially fatal complication [6–8]. Many researchers have reported the morbidity and mortality of SLE extensively; however, the pathogenic mechanism of SLE remains still elusive. Reports have indicated that autoantibody-mediated immune response can trigger tissue damage, and thus contributes to the pathogenesis of SLE [1, 9, 10]. In recent years, accumulating evidence indicates that deregulated production of proinflammatory cytokines such as TNF-*α* and IL-6 may play a critical role in immune dysfunction and mediate tissue inflammation and organ damage in SLE [1, 10]. It is reported that TNF-*α* levels are significantly increased and correlated with SLE disease activity, and blocking TNF-*α* function has been found to decrease disease activity in clinical patients [11–19]. Additionally, reports also indicate that IL-6 promotes autoantibody production in humans and mice with lupus nephritis [5, 20]. Therefore, the understanding of the detailed mechanism of inflammatory response would facilitate the advance of efficient therapies toward SLE.

Recent evidence indicates that HMGB1, a well-established damage associated molecular pattern (DAMP), is responsible for the production of proinflammatory cytokines [21–23]. HMGB1 is likely to be released from activated immune cells such as macrophages in the area of inflammation or injure [24–29]. When released, HMGB1 participates in the secretion of downstream proinflammatory cytokines via binding to cell surface receptors such as receptor of advanced glycation end products (RAGE), TLR2 and TLR4, thus contributing to the occurrence and development of diverse inflammatory diseases and autoimmune diseases [25–30]. Proinflammatory and immune-stimulatory function of HMGB1 indicate its association with autoimmune diseases including rheumatoid arthritis and SLE [29, 31]. Furthermore, HMGB1 has been found to be significantly elevated in lupus sera and identified as one of the components in DNA-containing immune complexes that enhance proinflammatory cytokine production [32]. All these data indicate that HMGB1 might act as a new inflammation-related factor in SLE; however, the precise role of HMGB1 in the inflammatory response during the pathogenesis of SLE still remains unclear.

Murine lupus model provides a good tool to investigate the pathogenesis of SLE. Our previous study has demonstrated that activated lymphocyte derived-DNA (ALD-DNA) could induce SLE syndrome including high levels of anti-dsDNA antibody, glomerulonephritis, and proteinuria in healthy mice with conventional genetic background [33–39]. In this study we investigated the potential role of HMGB1 in the pathogenesis of SLE and its underlying mechanism. We found that HMGB1 levels were elevated and correlated with SLE disease activity both in clinical patients and murine model. Gain- and loss-of-function analysis revealed that HMGB1 aggravated the severity of SLE, which might be due to its effect on macrophage inflammatory response. Furthermore, our findings showed that HMGB1-enhanced macrophage inflammatory response was dependent on RAGE.

## 2. Materials and Methods

### 2.1. Patients and Healthy Controls

The case-control study was approved by the Ethics Committee of Fudan University. A total of 32 SLE patients were recruited, and all of the peripheral blood samples were collected from these SLE patients after obtaining informed consent. The diagnosis of SLE was established according to the four of the American College of Rheumatology (ACR) revised criteria for the diagnosis of SLE. Disease activity was evaluated using SLEDAI. Lupus nephritis was diagnosed with renal biopsy. Patients who had other autoimmune diseases were excluded. Disease activity at the time of blood sampling was assessed by the SLEDAI. Further characteristics of the patients are summarized in Table 1. The mean age of the patients was 32 (range 19 to 54) years (y), and 24 healthy individuals matched for gender and age were recruited as controls.

### 2.2. Mice and Cell Culture

Six-week-old female BALB/c mice were purchased from the Experimental Animal Center of Chinese Academy of Sciences (Shanghai, China). Mice were housed in a specific pathogen free room under controlled temperature and humidity. This study was strictly carried out according to the Guide for the Care and Use of Medical Laboratory Animals (Ministry of Health, China, 1998) and with the ethical approval of the Shanghai Medical Laboratory Animal Care and Use Committee as well as the Ethical Committee of Fudan University. All surgery was performed under sodium pentobarbital anesthesia, and all animal procedures in this study were strictly performed in a manner to minimize suffering of laboratory mice. RAW264.7 cells were maintained in DMEM (Invitrogen Life Technologies) supplemented with 10% FBS (Invitrogen Life Technologies) in a 5% CO_2_ incubator at 37°C.

### 2.3. Reagents and Antibodies

pCAGGS-HMGB1 (pHMGB1) and pCAGGS (empty vector) were kindly provided by Professor Tadatsugu Taniguchi (University of Tokyo, Tokyo, Japan) [40]. HMGB1 blocker glycyrrhizin was purchased from Sigma. Glycyrrhizin was dissolved with PBS. TLR2/4 inhibitor OxPAPC was purchased from invivogen. RAGE-Fc was purchased from R&D Systems. The RAGE, HMGB1, and control siRNA were purchased from Santa Cruz Biotechnology. Macrophages were transfected with 200 nM of indicated siRNAs by Mouse Macrophage Nucleofector Kit (Lonza) according to the manufacturer's instructions. HMGB1 and RAGE antibody were obtained from Cell Signaling Technology and GAPDH antibody from Santa Cruz Biotechnology.

### 2.4. DNA Preparation and Generation of Murine Model of SLE

The extraction and purification of activated lymphocyte-derived DNA (ALD-DNA) and unactivated lymphocyte-derived DNA (UnALD-DNA) were performed according to our previously described methods [33–39]. To generate the murine model of SLE, six-week-old female BALB/c mice were immunized s.c. with ALD-DNA (50 *μ*g/mouse) plus CFA (Sigma-Aldrich) on day 1, followed by s.c. injection of ALD-DNA (50 *μ*g/mouse) emulsified with CFA (Sigma-Aldrich) on days 14 and 28 for total of three times as described previously [33–39]. Mice in each group received an equal volume of PBS plus CFA or IFA, or UnALD-DNA (50 mg/mouse) plus CFA or IFA were used as controls. To investigate the effect of HMGB1 in the pathogenic process of SLE, mice were injected intramuscularly with pHMGB1 or vector every two weeks. Mice were divided into six groups as follows: PBS plus vector, Un-ALD-DNA plus vector, ALD-DNA plus vector, PBS plus pHMGB1, Un-ALD-DNA plus pHMGB1 and ALD-DNA plus pHMGB1. To further confirm the significance of HMGB1, mice were treated with HMGB1 inhibitor glycyrrhizin (0.5 mg/mice) every day. Mice were divided into six groups as follows: PBS, Un-ALD-DNA plus PBS, ALD-DNA plus PBS, PBS plus glycyrrhizin, Un-ALD-DNA plus glycyrrhizin, and ALD-DNA plus glycyrrhizin. Serum and urine samples were collected every 2 weeks for further experiments. Eight weeks later, mice were sacrificed, and surgically resected kidneys were collected for further cellular function and tissue histology analysis.

### 2.5. pHMGB1 and Glycyrrhizin Treatment in Mice

To examine the potential role of HMGB1 in SLE, 8 mice in each group were intramuscularly injected with 100 *μ*g of pHMGB1 or empty vector per mouse 72 h earlier before injection with ALD-DNA. Mice were then injected with pHMGB1 every 2 weeks for total 6 times [35, 36]. To block the function of HMGB1 in SLE mice, mice were randomized to intramuscularly injection 0.5 mg per mouse glycyrrhizin every day for 2 months. Twenty-four hours after the initial glycyrrhizin treatment, the mice were immunized with ALD-DNA (50 *μ*g/mouse) three times in 4 week as previously described.

### 2.6. Anti-dsDNA Antibody and Proteinuria Examination

Serum anti-dsDNA antibody levels in the mice were determined by ELISA analysis as described previously [33–39]. Proteinuria of the mice was measured with the BCA method (Thermo Fisher Scientific) as previously described [33–39].

### 2.7. Cell Sorting

Murine renal tissues were surgically resected and dispersed in RPMI 1640 containing 5% FBS and 0.1% collagenase (Sigma-Aldrich) at 37°C for 30 min, followed by progressive sieving to obtain single-cell suspensions. To analyze the inflammatory response of renal macrophages, CD11b^+^/F4/80^high^ renal macrophages were sorted from nephritic single-cell suspensions using a FACSAria (BD Biosciences) with FITC-labeled anti-F4/80 and PE-labeled anti-CD11b (BD Biosciences).

### 2.8. Pathological Analysis

For histology analysis, murine renal tissues were surgically resected and fixed in 4% paraformaldehyde (Sigma-Aldrich), processed, and embedded in paraffin. H&E staining of renal tissue sections were performed according to the manufacturer's instructions and assessed by two pathologists blinded to treatment group as previously described [33–39]. The kidney score of glomerulonephritis was determined by using the ISN/RPS2003 classification. Pictures were acquired with Nikon SCLIPSS TE2000-S microscope (Nikon) equipped with ACT-1 software (Nikon).

### 2.9. ELISA

Plasma was collected by EDTA as an anticoagulant, aliquoted, and stored at −80°C. To assess the levels of HMGB1, anti-dsDNA antibody, TNF-*α*, and IL-6 in the plasma and supernatant of cell culture, ELISA (Shino-Test, Sagamihara-shi, Kanagawa, Japan for HMGB1; ebioscience for TNF-*α* and IL-6; Alpha Diagnostic International for anti-dsDNA) was performed according to the manufacturer's instructions.

### 2.10. Western Blot

Western blot was performed as described previously [33–39]. Antibodies used here were anti-GAPDH (Santa Cruz Biotechnology), HMGB1 (Cell Signal Technology), goat anti-mouse IgG-HRP (Santa Cruz Biotechnology), and goat anti-rabbit IgG-HRP (Santa Cruz Biotechnology).

### 2.11. Statistical Methods

Data was represented as the mean ± standard deviation (SD). Comparisons between SLE patients and HC were analyzed by Student's *t* test. Correlation analysis was performed by Pearson correlation test. All analyses were performed by GraphPad Prism 5 (GraphPad Software, La Jolla, CA). A two-tailed *P* value < 0.05 was considered as statistically significant unless otherwise noted.

## 3. Results

### 3.1. Serum HMGB1 Levels Were Elevated and Correlated with SLE Disease Activity Both in Clinical Patients and Murine Model

**Figure 1:**
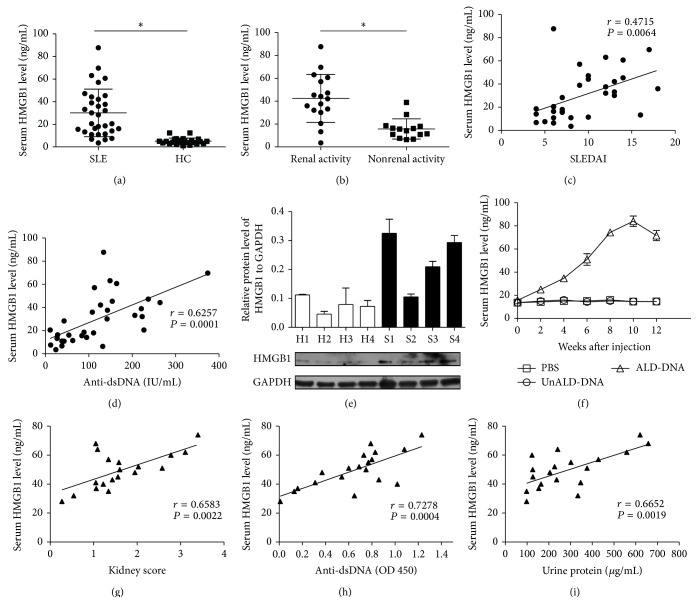
HMGB1 levels were elevated and correlated with SLE disease activity in both clinical patients and murine model. (a) Serum HMGB1 levels were detected by ELISA in SLE patients and HC. (b) HMGB1 levels were measured by ELISA in renal active or inactive patients. The scatter-plot represented the HMGB1 levels by ELISA. Each symbol represents one SLE patient. Horizontal lines represent the median. Data represent the average from experiments performed in triplicates for each patient. (c) Correlation analysis was performed between HMGB1 levels and SLEDAI. (d) Correlation analysis was performed between HMGB1 and anti-dsDNA antibody levels. Pearson correlation analysis was used in the correlation analysis. (e) The expression of HMGB1 was analyzed by western blot in PBMCs from SLE patients (S) and healthy controls (H). Representative western blot bands from 4 patients with SLE and 4 HC were presented. Data were representative of results obtained in three independent experiments. (f) Serum HMGB1 levels were measured by ELISA every 2 weeks after initial injection. Data are means ± SD from 8 mice in each group. (g) The correlation between serum HMGB1 levels and kidney score was carried out in SLE mice. (h) The correlation between serum HMGB1 and anti-dsDNA antibody levels was carried out in SLE mice. (i) The correlation between serum HMGB1 and urine protein levels was carried out in SLE mice. Pearson correlation analysis was used to carry out the correlation study. Each symbol indicates an individual mouse (*n* = 19). ^*∗*^ *P* < 0.05.

To investigate whether HMGB1 was involved in the pathogenesis of SLE, we first examined the levels of HMGB1 in SLE patients. A total of 32 SLE patients were recruited to our research and the general characteristics of patients were shown in Table 1. We determined the serum concentrations of HMGB1 in SLE patients and healthy controls (HC) by ELISA. The results showed that HMGB1 concentrations in SLE patients (30.1356 ± 21.0236 ng/mL) were significantly higher than those in HC (5.0877 ± 2.7921 ng/mL, *P* < 0.05) (Figure 1(a)). Furthermore, serum HMGB1 levels in SLE patients with active renal disease (42.5672 ± 21.0052 ng/mL) were significantly higher than those in patients without active renal disease (15.7279 ± 8.8412 ng/mL, *P* < 0.05, Figure 1(b)). Moreover, we found that serum HMGB1 concentrations showed a highly significant correlation with SLE disease activity index score (SLEDAI) (*r* = 0.4715, *P* = 0.0064, Figure 1(c)) and anti-dsDNA antibody levels (*r* = 0.6257, *P* = 0.0001, Figure 1(d)). We also analyzed the expression of HMGB1 in peripheral blood mononuclear cells (PBMCs) from SLE patients (S1, S2, S3, and S4) and HC (H1, H2, H3, and H4) using western blot. As shown in Figure 1(e), the expression of HMGB1 was increased in PBMCs from SLE patient compared to HC.

We further analyzed the serum levels of HMGB1 in murine model of SLE which were generated according to our previously reported procedures [33–39]. ALD-DNA could successfully induce the SLE syndrome manifested by high levels of anti-dsDNA antibody (see Figure S1(a) in Supplementary Material available online at http://dx.doi.org/10.1155/2015/946748), proteinuria (Figure S1(b)) as well as glomerulonephritis (Figures S1(c) and S1(d)). Of interest, we found that serum HMGB1 levels were significantly increased in murine model of SLE compared with those in control mice (Figure 1(f)). Pearson correlation analysis showed that the serum HMGB1 levels were positively correlated with kidney score (*r* = 0.6583, *P* = 0.0022, Figure 1(g)), indicating that HMGB1 levels were significantly associated with the severity of lupus nephritis. Similarly, we observed that serum HMGB1 levels were related with the levels of anti-dsDNA antibody (*r* = 0.7278, *P* = 0.0004, Figure 1(h)) and urine protein (*r* = 0.6652, *P* = 0.0019, Figure 1(i)) in ALD-DNA-induced murine model of SLE. Taken together, these data indicated that HMGB1 expression was upregulated and correlated with the severity of SLE both in clinical patients and murine model.

### 3.2. Forced/Inhibited HMGB1 Expression Modulated the Severity of SLE

**Figure 2:**
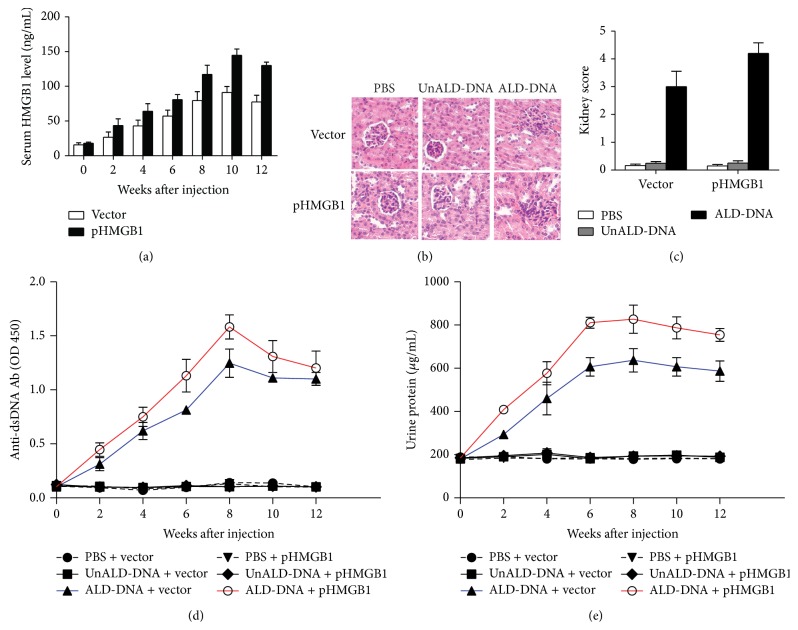
HMGB1 overexpression could promote the severity of SLE. BALB/c mice were administrated intramuscularly with 100 *μ*g pHMGB1 or empty-vector per mouse. 72 h following injection, mice were then injected subcutaneously with ALD-DNA (50 *μ*g/mouse) for total 3 times in 4 weeks. (a) The dynamics of serum HMGB1 levels were determined by ELISA every 2 weeks after initial injection. Data are means ± SD from 8 mice in each group. (b) Nephritic pathology was evaluated by H&E staining of renal tissues. Images (magnification ×200) are representative of at least 8 mice in each group. (c) The kidney score was assessed using paraffin sections stained with H&E in (b). (*n* = 8). (d) Serum anti-dsDNA antibody levels were measured by ELISA every 2 weeks after initial injection. Data are means ± SD from 8 mice in each group. (e) Urine protein levels of the mice were assessed by BCA method every 2 weeks. Data are means ± SD from 8 mice in each group. ^*∗*^ *P* < 0.05.

Above data showed that HMGB1 levels were elevated and correlated with the severity of SLE. To further evaluate whether HMGB1 was involved in the pathogenesis of SLE, we upregulated the expression of HMGB1 by injecting BALB/c mice intramuscularly with a HMGB1 overexpression plasmid (pHMGB1). Results showed that the injection of pHMGB1 led to the elevation of serum HMGB1 levels in SLE mice (Figure 2(a)). To investigate the effect of increased HMGB1 levels on the progression of SLE, we analyzed anti-dsDNA antibody, proteinuria, renal pathology, and kidney score in pHMGB1- or empty vector-treated mice. Results showed that HMGB1 overexpression exacerbated renal pathology as revealed by the increased infiltration of proinflammatory cells into glomerular mesanguim and thickened basement membrane, as well as the atrophy of glomerular mesanguim (Figure 2(b)), and upregulated the kidney score of SLE mice (Figure 2(c)). We also found that the levels of anti-dsDNA antibody (Figure 2(d)) and urine protein (Figure 2(e)) in SLE mice treated with pHMGB1 were notably elevated compared with that in empty vector-treated SLE mice.

**Figure 3:**
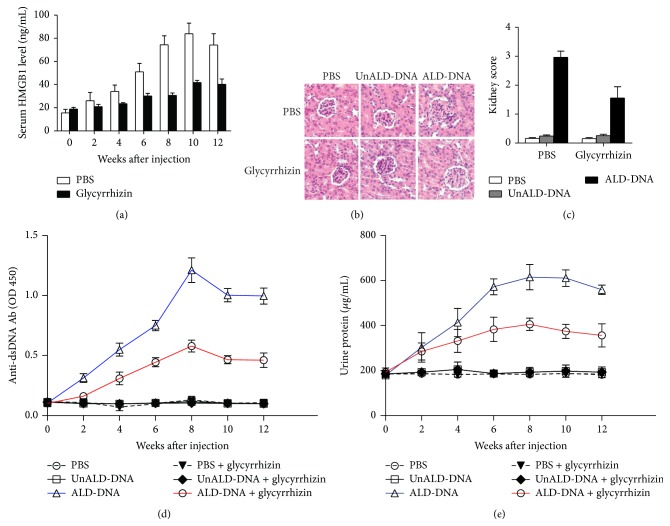
Inhibition of HMGB1 function could ameliorate the severity of SLE. BALB/c mice were administrated intramuscularly injected with glycyrrhizin (0.5 mg/mouse) or PBS to inhibit HMGB1 function. 72 h after injection, mice were then injected subcutaneously with ALD-DNA (50 *μ*g/mouse) for total 3 times in 4 weeks. (a) The dynamics of serum HMGB1 levels were determined by ELISA every 2 weeks after initial injection. Data are means ± SD from 8 mice in each group. (b) Nephritic pathology was evaluated by H&E staining of renal tissues. Images (magnification ×200) are representative of at least 8 mice in each group. (c) The kidney score was assessed using paraffin sections stained with H&E in (b). (*n* = 8). (d) Serum anti-dsDNA antibody levels were measured by ELISA every 2 weeks after initial injection. Data are means ± SD from 8 mice in each group. (e) Urine protein levels of the mice were assessed by BCA method every 2 weeks. Data are means ± SD from 8 mice in each group. ^*∗*^ *P* < 0.05.

To further confirm the effect of HMGB1 on the progression of SLE, we inhibited the function of HMGB1* in vivo* by injecting BALB/c mice intramuscularly with glycyrrhizin which has been demonstrated to be the blocker of HMGB1 [41–43]. As shown in Figure 3(a), glycyrrhizin administration significantly decreased the serum HMGB1 levels in SLE mice. Of note, glycyrrhizin treatment was found to efficiently ameliorate renal pathology as demonstrated by decreased infiltration of proinflammatory cells into glomerular mesanguim, recovery from thicken basement membrane and the atrophic glomerular mesanguim (Figure 3(b)), and decrease the kidney score in SLE mice (Figure 3(c)). Furthermore, we revealed that glycyrrhizin treatment reduced levels of anti-dsDNA antibody (Figure 3(d)) and urine protein (Figure 3(e)) in SLE mice.

Taken together, these data demonstrated that HMGB1 played a crucial role in modulating the severity of SLE.

### 3.3. HMGB1 Enhanced Macrophage Inflammatory Response and Corresponded to the Proinflammatory Cytokines in SLE

**Figure 4:**
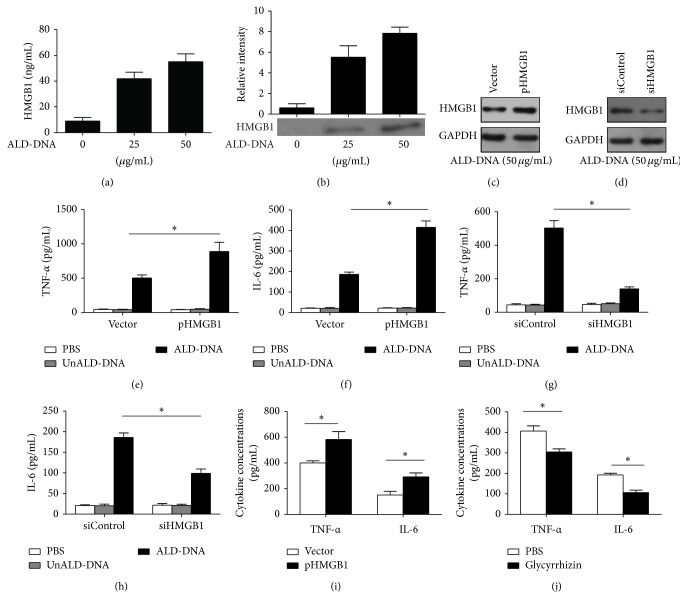
HMGB1 aggravated macrophage inflammatory response. (a-b) RAW264.7 cells were stimulated with ALD-DNA (0, 25, 50 *μ*g/mL) for 24 h, levels of HMGB1 in the supernatants of RAW264.7 cells were analyzed by ELISA (a) and western blot analysis (b). Data are means ± SD of three independent experiments. (c-d) The efficiency of HMGB1 overexpression (c) and knockdown (d) was monitored by representative immunoblot of three independent experiments in ALD-DNA-stimulated RAW264.7 cells. (e-f) RAW264.7 cells were transfected with pHMGB1 or vector. 72 h after transfection, RAW264.7 cells were stimulated with PBS, UnALD-DNA or ALD-DNA (50 *μ*g/mL) followed by analyzing the levels of TNF-*α* (e) and IL-6 (f) in the culture supernatants of RAW264.7 cells. Data are means ± SD of three independent experiments. (g-h) RAW264.7 cells were transfected with control siRNA (200 nM) or HMGB1 siRNA (siHMGB1, 200 nM). After 72 h RAW264.7 cells were stimulated with PBS, UnALD-DNA or ALD-DNA (50 *μ*g/mL). ELISA assay was used to analyze the levels of TNF-*α* (g) and IL-6 (h) in the culture supernatants of RAW264.7 cells. Data are means ± SD of three independent experiments. (i-j) CD11b^+^/F4/80^high^ renal macrophages were sorted from nephritic single-cell suspensions from (i) pHMGB1- or empty vector-treated, (j) glycyrrhizin- or PBS-treated SLE mice by flow cytometry. Macrophages (2 × 10^5^/mL) were stimulated with ALD-DNA (50 *μ*g/mL) for 24 h. The supernatants were collected and assayed for the TNF-*α* and IL-6 concentrations using ELISA. Data are means ± SD from 8 mice in each group. ^*∗*^ *P* < 0.05.

It is well established that macrophages are prominent within the inflamed kidneys and are key mediators in lupus nephritis [44–49]. Our previous studies have also confirmed that macrophage is the central mediator in ALD-DNA-induced SLE [33–36]. To study whether HMGB1 was involved in macrophage inflammatory response, we detected the production of HMGB1 in RAW264.7 cells stimulated with ALD-DNA. The results showed that the ALD-DNA administration led to the upregulation of HMGB1 levels in RAW264.7 cells (Figures 4(a) and 4(b)). We then investigated the role of HMGB1 in ALD-DNA-induced macrophage inflammatory response by transfecting pHMGB1 into RAW264.7 cells. Results showed that transfection of pHMGB1 notably increased HMGB1 levels in RAW264.7 cells (Figure 4(c)). Of importance, HMGB1 overexpression in RAW264.7 cells was found to aggravate the secretion of TNF-*α* (Figure 4(e)) and IL-6 (Figure 4(f)) induced by ALD-DNA. We further downregulated HMGB1 expression by specific siRNA against HMGB1 (siHMGB1) in RAW264.7 cells. Western blot showed that transfection of siHMGB1 efficiently decreased HMGB1 levels in RAW264.7 cells (Figure 4(d)). We stimulated siHMGB1-treated RAW264.7 cells with ALD-DNA, followed by detecting the concentrations of proinflammatory cytokines in the supernatants. Results showed that the siHMGB1-mediated downregulation of HMGB1 levels significantly inhibited the secretion of TNF-*α* (Figure 4(g)) and IL-6 (Figure 4(h)) in ALD-DNA-stimulated macrophages. Moreover, we isolated CD11b^+^/F4/80^high^ renal macrophages from pHMGB1- or empty vector-treated SLE mice, glycyrrhizin- or PBS-treated SLE mice and stimulated these cells with ALD-DNA, followed by detecting the production of TNF-*α* and IL-6. As shown in Figure 4(i), renal macrophages secreted much higher levels of TNF-*α* and IL-6 in pHMGB1-treated SLE mice than those from empty vector-treated SLE mice, whereas renal macrophages from glycyrrhizin-treated SLE mice secreted lower levels of TNF-*α* and IL-6 than those PBS-treated SLE mice (Figure 4(j)).

**Figure 5:**
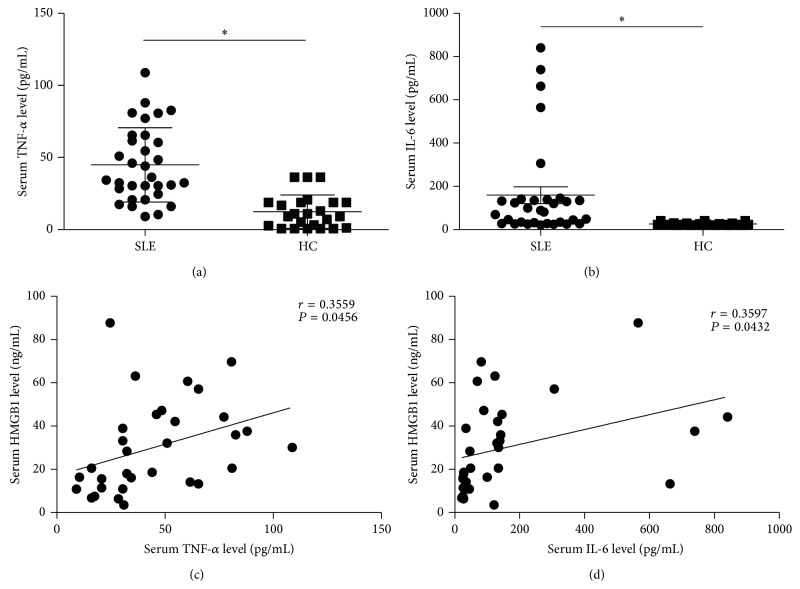
The HMGB1 levels were associated with proinflammatory cytokines in SLE patients. (a-b) TNF-*α* (a) and IL-6 (b) concentrations in sera from SLE patients and HC were detected by ELISA. The scatter-plot represented the TNF-*α* and IL-6 levels by ELISA analysis. Each symbol represents one SLE patient. Horizontal lines represent the median. Data represent the average from experiments performed in triplicates for each patient. (c-d) Correlation analyses were presented between HMGB1 and TNF-*α* levels (c), HMGB1 and IL-6 levels (d). Pearson correlation analysis was used in the correlation analysis. ^*∗*^ *P* < 0.05.

To study the relationship between HMGB1 and proinflammatory cytokines, we analyzed the correlation between HMGB1 and proinflammatory cytokines (TNF-*α* and IL-6) in SLE patients. We first detected the levels of serum TNF-*α* and IL-6 in SLE patients, and the results demonstrated that the concentrations of TNF-*α* (Figure 5(a)) and IL-6 (Figure 5(b)) in SLE patients were significantly higher than those in HC (*P* < 0.05). Further study was conducted to analyze the correlation between HMGB1 and proinflammatory cytokines (TNF-*α* and IL-6). We observed that HMGB1 levels were associated with TNF-*α* (*r* = 0.3559, *P* = 0.0456, Figure 5(c)) and IL-6 levels (*r* = 0.3597, *P* = 0.0432, Figure 5(d)) in SLE patients.

Taken together, these data indicated that HMGB1 was pivotal for ALD-DNA-induced macrophage inflammatory response and correlated with the levels of TNF-*α* and IL-6 in SLE.

### 3.4. HMGB1-Enhanced Macrophage Inflammatory Response Was Dependent on RAGE but Not on TLR2 and TLR4

**Figure 6:**
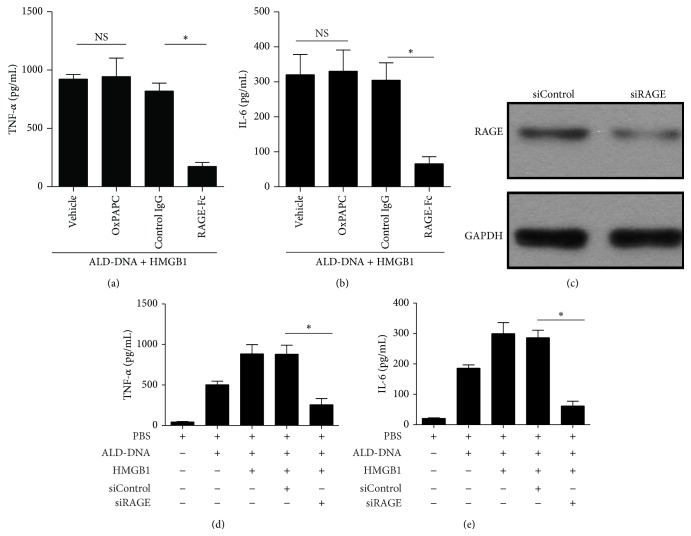
HMGB1-enhanced macrophage inflammatory response induced by ALD-DNA might be dependent on RAGE but not on TLR2 and TLR4. (a-b) RAW264.7 cells were transfected with pHMGB1, and then stimulated with ALD-DNA (50 *μ*g/mL) in the presence of OxPAPC (30 *μ*g/mL) or RAGE-Fc (10 *μ*g/mL) for 24 h. The supernatants were collected and assayed for the concentrations of TNF-*α* (a) and IL-6 (b) using ELISA. (c) Representative immunoblot of three independent experiments has shown the efficiency of RAGE knockdown. (d-e) RAW264.7 cells transfected with siRAGE and pHMGB1 were stimulated with 50 *μ*g/mL of ALD-DNA for 24 h. The supernatants were collected and assayed for the concentrations TNF-*α* (d) and IL-6 (e) using ELISA.^*∗*^ *P* < 0.05.

Previous studies indicate that HMGB1 is an endogenous ligand of RAGE, TLR2, and TLR4 [50–52]. To evaluate which receptor might be involved in the HMGB1-mediated inflammatory effect in ALD-DNA-stimulated macrophages, we first upregulated HMGB1 expression in RAW264.7 cells and then stimulated these cells with ALD-DNA in the presence of TLR2/4 inhibitor (OxPAPC) or RAGE inhibitor (RAGE-Fc). The results showed that the production of TNF-*α* (Figure 6(a)) and IL-6 (Figure 6(b)) from RAW264.7 cells exposed to TLR2/4 inhibitor was not impaired in the process of HMGB1-enhanced macrophage inflammatory response, whereas blocking the function of RAGE with RAGE-Fc effectively reduced the secretion of TNF-*α* (Figure 6(a)) and IL-6 (Figure 6(b)). To further validate the importance of RAGE receptor, we downregulated the expression of RAGE by siRNA and then evaluated the effect of HMGB1 on the production of proinflammatory cytokines in ALD-DNA-stimulated RAW264.7 cells. Western blot analysis confirmed that transfection of siRAGE could notably decrease RAGE levels in RAW264.7 cells (Figure 6(c)). Of importance, it was found that siRNA-mediated downregulation of RAGE significantly inhibited HMGB1-enhanced release of TNF-*α* (Figure 6(d)) and IL-6 (Figure 6(e)) in ALD-DNA-stimulated macrophages. Collectively, these data indicated that RAGE might be critical for proinflammatory signaling during the process of HMGB1-aggravated macrophages inflammatory response induced by ALD-DNA, whereas TLR2 and TLR4 appeared to be dispensable.

## 4. Discussion

SLE, a chronic inflammatory autoimmune disorder, is a potentially fatal disease characterized by immune complex deposition and the subsequent inflammation that contributes to severe tissue damage [3, 5]. Recent reports show that some multifunctional proteins such as HMGB1 might be involved in autoimmune and inflammatory diseases including SLE [31, 52–55]. Our previous study revealed that HMGB1 was required for autoantibody induction by DNA-containing immune complexes in SLE patients [56], implying that the critical role of HMGB1 in the pathogenesis of SLE. In the present investigation, we further explored the role and mechanism of HMGB1 in the pathogenesis of SLE.

Our results demonstrated that HMGB1 levels were increased and correlated with the severity of SLE in both clinical patients and murine model, consistent with previous findings [57–60]. To evaluate whether HMGB1 was involved in the pathogenesis of SLE, we overexpressed HMGB1 in ALD-DNA-immunized mice and found that HMGB1 overexpression significantly enhanced the severity of SLE. To further confirm the role of HMGB1 in SLE, we broke its function with glycyrrhizin. Glycyrrhizin is commonly used in clinical application for its anti-inflammatory activity, and it binds to HMGB1 directly, thereby inhibiting the extracellular HMGB1 secretion [41]. Evidence also shows that glycyrrhizin administration could suppress HMGB1 function resulting in the amelioration of ischemic spinal cord injury and damage caused by cerebral hemorrhage [42, 43]. Our results showed that blockade of HMGB1 function by glycyrrhizin led to dramatic downregulation of serum HMGB1 levels, and thus decreased the severity of SLE. These data suggested that HMGB1 played a crucial role in the pathogenesis of SLE, implicating a promising HMGB1-based therapeutic strategy against SLE. However, the mechanisms of HMGB1 elevation in SLE still deserved further studies.

The increasing number of evidence has emerged to suggest the crucial role of proinflammatory cytokines in the pathogenesis of SLE. The consequence of disorder of proinflammatory cytokines would be an immune dysregulation followed by local inflammatory processes and tissue damage [61, 62]. Circumstantial data suggests that TNF-*α* may serve as an important autocrine and paracrine factor in glomerular injury [11–13]. In addition, IL-6 is produced in many cell types like monocytes, fibroblasts, endothelial cells, and also T and B lymphocytes and has a range of biological activities on various target cells [63]. Considering the importance of HMGB1 in the regulation of inflammatory response, we analyzed the correlation between HMGB1 and proinflammatory cytokines expression in SLE. Our studies found that the concentrations of TNF-*α* and IL-6 in SLE patients were significantly higher than those in HC, consistent with previous study [64]. Further studies showed that HMGB1 levels were correlated with the levels of TNF-*α* and IL-6 in SLE patients. Moreover, our data found that HMGB1 promoted inflammatory response of renal macrophages in SLE mice. These results suggested that HMGB1 might be involved in the pathogenesis of SLE via regulating macrophage inflammatory response, however, the definite relationship between HMGB1 and macrophage inflammatory response needs further investigation.

Accumulating data demonstrate that activated macrophages that infiltrate kidneys mediate the onset of an aggressive adaptive immune response leading to the pathogenesis of SLE in mice [65–70]. These reports give a clue that macrophages play a crucial pathogenic role in the development of SLE. Our previous study has also indicated that ALD-DNA immunization lead to macrophage infiltration and aberrant activation, which mediate the onset and aggravation of SLE, indicating that aberrant activation of macrophage plays a crucial pathogenic role in ALD-DNA-induced SLE [33–39]. Here, we found that HMGB1 enhanced ALD-DNA-induced macrophage inflammatory response both* in vivo* and* in vitro*. The HMGB1 levels were closely correlated with macrophage inflammatory response. HMGB1 is a ubiquitously expressed, abundant architectural chromosomal protein of 215 amino acids, with a highly conserved sequence across species [29]. At least three receptors are reported to mediate the proinflammatory and immune-activate effects of extracellular HMGB1: RAGE, TLR2, and TLR4 [25–28]. Our results suggested that RAGE might be critical for proinflammatory signaling during the process of HMGB1-aggravated macrophage inflammatory response induced by ALD-DNA, whereas TLR2 and TLR4 seemed to be dispensable. Collectively, it seemed that HMGB1 was a crucial cofactor that could modify the stimulatory activity of macrophage.

## 5. Conclusion

In summary, our research reported that HMGB1 levels were significantly increased and correlated with SLE disease activity in both clinical patients and murine model. Further study suggested that HMGB1 aggravated the severity of SLE via facilitating macrophage inflammatory response. Moreover, RAGE might be critical for proinflammatory signaling during the process of HMGB1-aggravated macrophages inflammatory response. These findings may help to develop anti-inflammatory therapeutics which blunted macrophage activation by blocking HMGB1 function in SLE.

## Supplementary Material

Figure S1: ALD-DNA immunization induces SLE syndrome.

## Figures and Tables

**Table 1:** Characteristics of systemic lupus erythematosus (SLE) patients and control subjects.

	SLE	Control
Number	32	24
Sex (female/male)	32/0	24/0
SLEDAI score mean ± s.d.	9.3 ± 3.9	n.a
Anti-dsDNA titre mean ± s.d. (IU/mL)	121.2 ± 86.1	n.a
Patients with nephritis (%)	(17/32) 53%	n.a
Treatment with prednisolone		
Patients, number (%)	93.75%	n.a
Treatment with hydroxychloroquine		
Patients, no. (%)	87.5%	n.a
Treatment with azathioprine		
Patients, no. (%)	0%	n.a

Values are in mean ± standard deviation (s.d.); n.a.: not applicable.
