# Supplementary material for: HMGB1 Promotes Systemic Lupus Erythematosus by Enhancing Macrophage Inflammatory Response
Source: J Immunol Res. 2015 May 19;2015:946748. doi: 10.1155/2015/946748 (PMC4452473; doi:10.1155/2015/946748)
Supplement: Supplementary file 1 — Figure S1: ALD-DNA immunization induces SLE syndrome. [file 946748.f1.pdf]

Supplementary Information

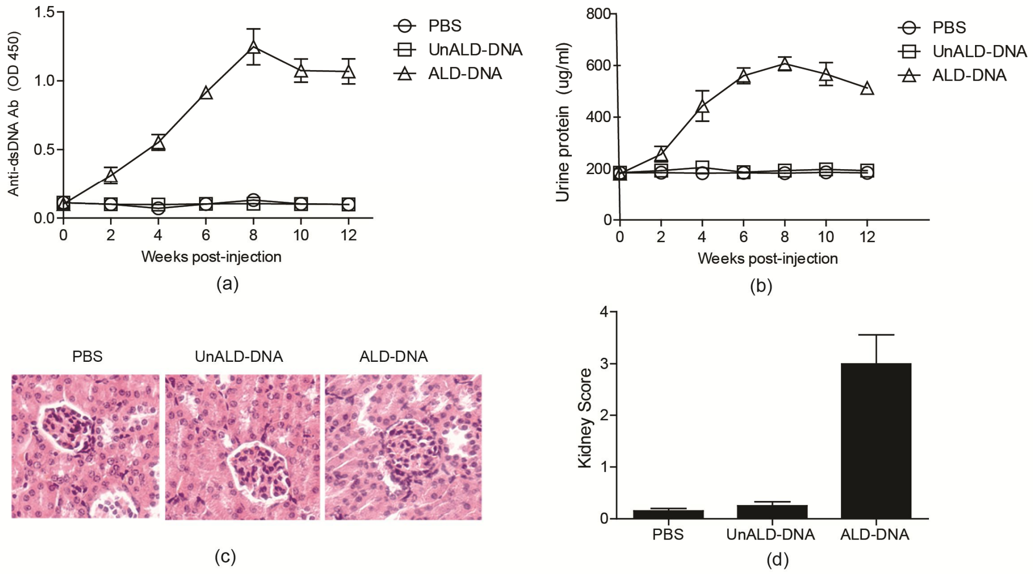

Figure S1 ALD-DNA immunization induces SLE syndrome. (a) Serum anti-dsDNA IgG levels were measured by ELISA every 2 weeks after initial injection. Data are means ± SD from 8 mice in each group. (b) Urine protein levels of the mice were assessed by BCA Protein Assay Kit every 2 weeks. Data are means ± SD from 8 mice in each group. (c) Nephritic pathology was evaluated by H&E staining of renal tissues. Images (magnification ×200) are representative of 8 mice in each group. (d) The kidney score was assessed using paraffin sections stained with H&E in (c). n=8.
